# Supplementary material for: Neprilysins regulate muscle contraction and heart function via cleavage of SERCA-inhibitory micropeptides
Source: Nat Commun. 2022 Jul 29;13:4420. doi: 10.1038/s41467-022-31974-1 (PMC9338278; doi:10.1038/s41467-022-31974-1)
Supplement: Supplementary file 1 — Supplementary Information [file 41467_2022_31974_MOESM1_ESM.pdf]

Supplementary Figures

Supplementary figure 1

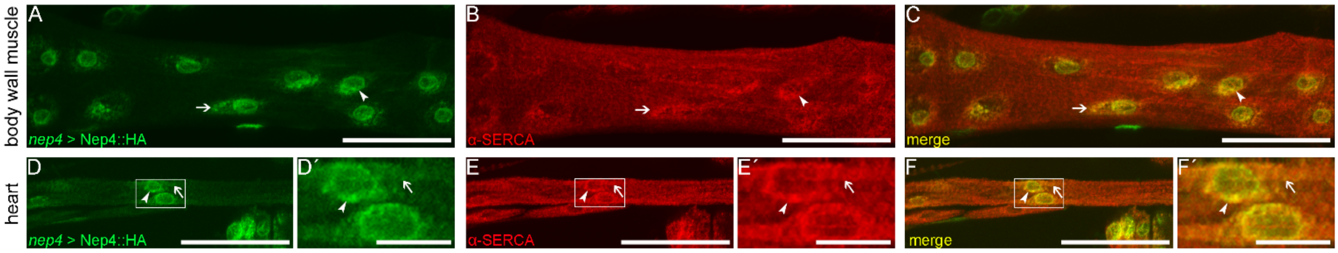

Nep4 partially colocalizes with SERCA in third instar larval heart and body wall muscles. (**A, D**) Nep4::HA protein was expressed under the control of the native *nep4* enhancer and labeled with a monospecific antibody against the HA-tag (*nep4* > Nep4::HA). (**B, E**) SERCA was labeled with a monospecific antibody detecting the endogenous protein (SERCA). Optical projections of a third instar larval body wall muscle fiber (**A-C**) or heart tube (**D-F**) are shown. Scale bars: 50 µm; ventral view, anterior left. Boxes indicate areas of higher magnification, as depicted in (**D´-F´**). Scale bars: 10 µm. Nep4::HA colocalizes with SERCA in membranes contiguous with the nuclear membrane (arrowheads). In addition, both proteins partially colocalize in a punctate manner along the muscle tissue (arrows).

Supplementary figure 2

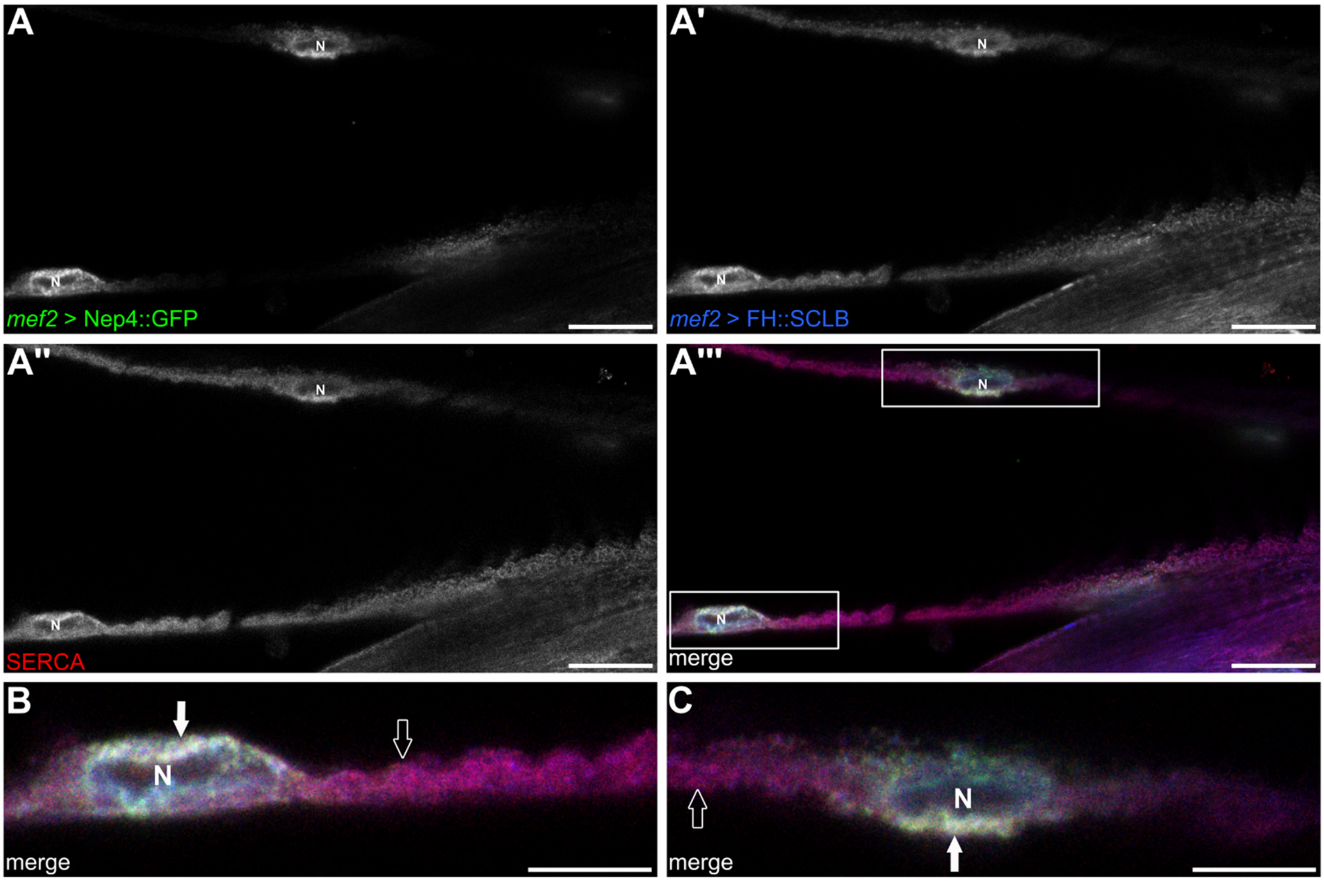

Nep4 partially colocalizes with Sarcolamban B in larval heart tissue.

GFP-tagged Nep4 (*mef2* > Nep4::GFP, **A**) and FH-tagged Sarcolamban B (*mef2* > FH::SCLB, **A´**) were expressed under the control of the muscle-specific *mef2* enhancer and labeled with monospecific antibodies against the GFP- or the FH-tag, respectively. SERCA was labeled with a monospecific antibody detecting the endogenous protein (SERCA, **A´´**). Colocalization of all three factors is visible around the nuclei (**B**, **C**, solid arrow). More distant from the nuclei, only SERCA and SCLB signals are present (**B**, **C**, open arrow). Confocal images of third instar larval heart tissue are shown. Scale bars: 20 µm; ventral view, anterior left. Insets indicate areas of higher magnification as depicted in **B** and **C**. Scale bars: 10 µm.

Supplementary figure 3

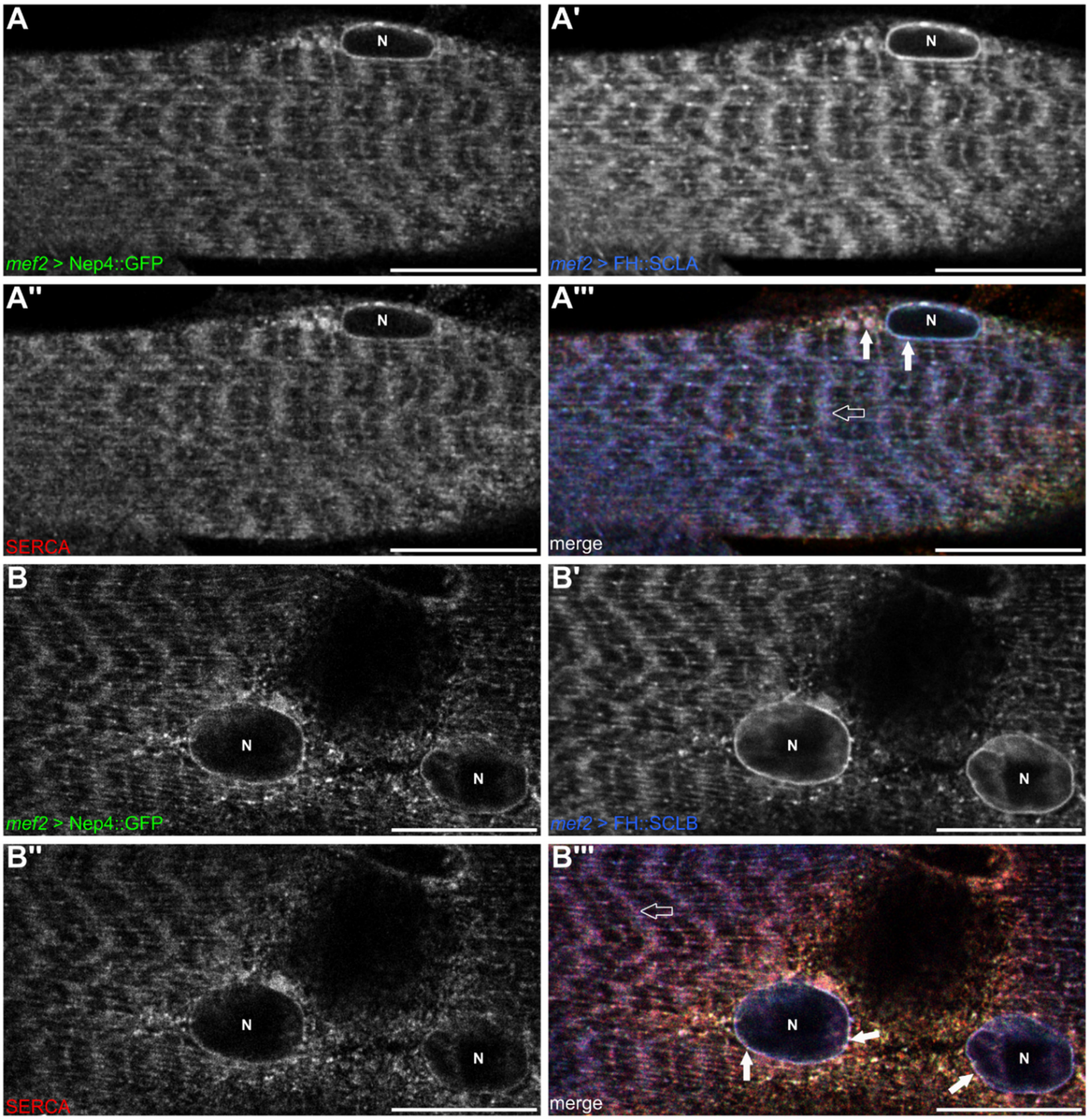

Nep4 partially colocalizes with SCLA and SCLB in larval body wall muscles.

GFP-tagged Nep4 (*mef2* > Nep4::GFP, **A, B**) and FH-tagged Sarcolamban A (*mef2* > FH::SCLA, **A´**) or Sarcolamban B (*mef2* > FH::SCLB, **B´**) were expressed under the control of the muscle-specific *mef2* enhancer and labeled with monospecific antibodies against the GFP- or the FH-tag, respectively. SERCA was labeled with a monospecific antibody detecting the endogenous protein (SERCA, **A´´**, **B´´**). Colocalization of Nep4, SCLA, and SERCA is visible around the nuclei (**A´´´**, solid arrows). More distant from the nuclei, only SERCA and SCLA signals are present (**A´´´**, open arrow). A similar localization pattern is apparent for Nep4, SCLB, and SERCA (**B´´´**). Confocal images of third instar larval body wall muscles are shown. Scale bars: 20 µm; ventral view, anterior left.

Supplementary figure 4

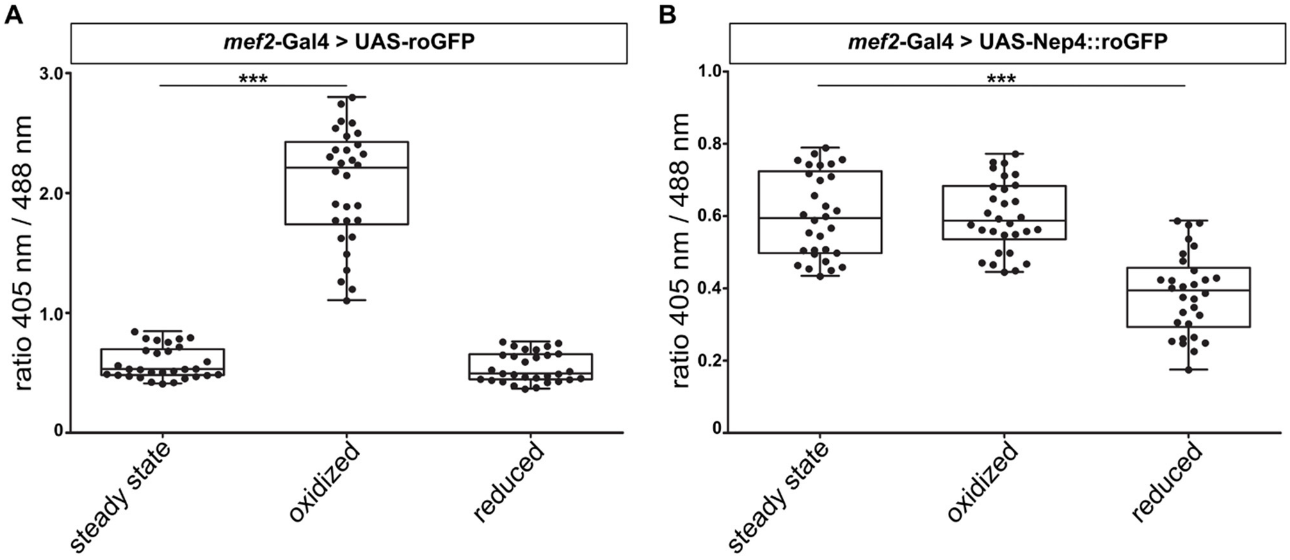

The C-terminus of Nep4 localizes to the SR lumen.

A Nep4::roGFP fusion (*mef2*-Gal4 > UAS-Nep4::roGFP) was employed to determine the orientation of the protein within the SR membrane. Free roGFP was used as a control (*mef2*-Gal4 > UAS-roGFP). While the steady-state fluorescence intensity ratio (405 nm / 488 nm excitation) of free roGFP reflects reducing conditions (**A**), roGFP fused to the C-terminus of Nep4 exhibits a corresponding ratio that is characteristic for oxidizing conditions (**B**). The center line of a boxplot indicates the median; the upper and lower bounds indicate the 75th and 25th percentiles, respectively; and the whiskers indicate the minimum and maximum. Asterisks indicate statistically significant deviations from respective controls ($p < 0.001$, one-way ANOVA followed by Dunnett's Multiple Comparison Test). n = 30 ROIs examined over 3 individual animals (10 ROIs per animal). Source data are provided as a Source Data file.

Supplementary figure 5

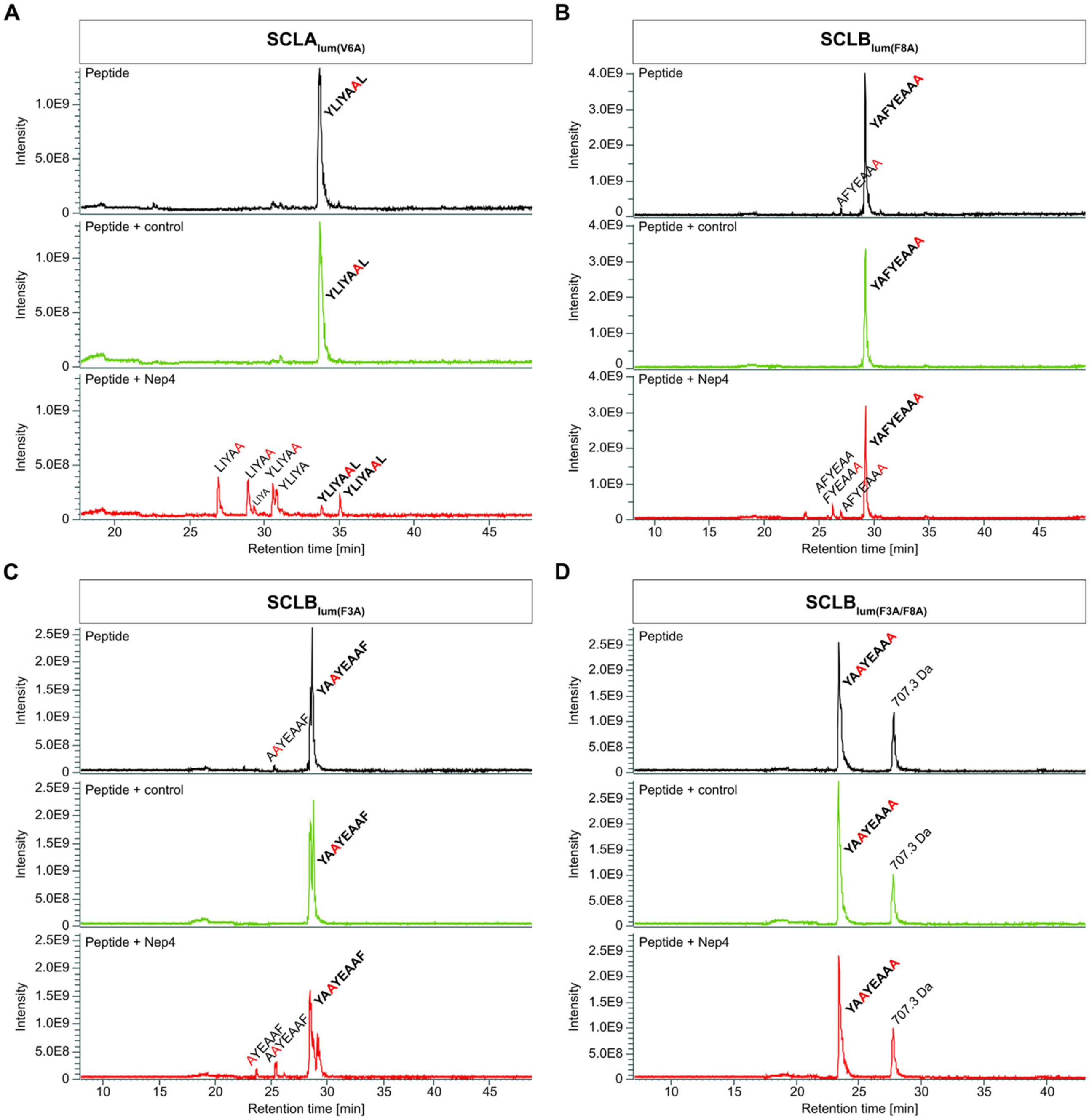

The amino acid at P1´ is a critical determinant of the Nep4 cleavage specificity.

Depicted are total ion chromatograms of mutated SCLA$_{lum}$ (**A**) and SCLB$_{lum}$ derivatives (**B**-**D**). Mutated residues are marked in red. Full-length peptides (bold) are detected under all applied experimental conditions (pure peptide, black chromatogram; peptide incubated with control preparation, green chromatogram; peptide incubated with purified Nep4, red chromatogram). Italicized fragments could not unambiguously be assigned to one distinct peptide sequence. (**A**) Substitution of Val-6 by Ala (SCLA$_{lum(V6A)}$, YLIYAAL) results in additional cleavage between Tyr-1 and Leu-2 as well as between Ala-6 and Leu-7, giving rise

to the peptide fragments LIYAA, LIYA and YLIYAA (red chromatogram). (**B**) The $SCLB_{lum(F8A)}$ derivative (YAFYEAAA) is largely resistant to Nep4-mediated hydrolysis (red chromatogram). Aside from the full-length peptide, only minor amounts of a AFYEAAA cleavage product are detected; a third peak of low amplitude corresponds to either *AFYEAA* or *FYEAAA* (See also Table S1). (**C**) The $SCLB_{lum(F3A)}$ derivative (YAAYEAAF) is largely resistant to Nep4-mediated hydrolysis (red chromatogram). Aside from the full-length peptide, only minor amounts of the fragments AYEAAF and AAYEAAF are detected, with the latter also being present in the untreated peptide sample (black chromatogram). (**D**) The $SCLB_{lum(F3A/F8A)}$ derivative (YAAYEAAA) is completely resistant to Nep4-mediated hydrolysis; no cleavage products are detected (red chromatogram). In addition to the full-length peptide (YAAYEAAA), under all three experimental conditions a non-assignable peak with a molecular mass of 707.3 Da is present. Y-axes show absolute peak intensities, X-axes depict retention times. Individual cleavage assays were repeated at least three times.

Supplementary figure 6

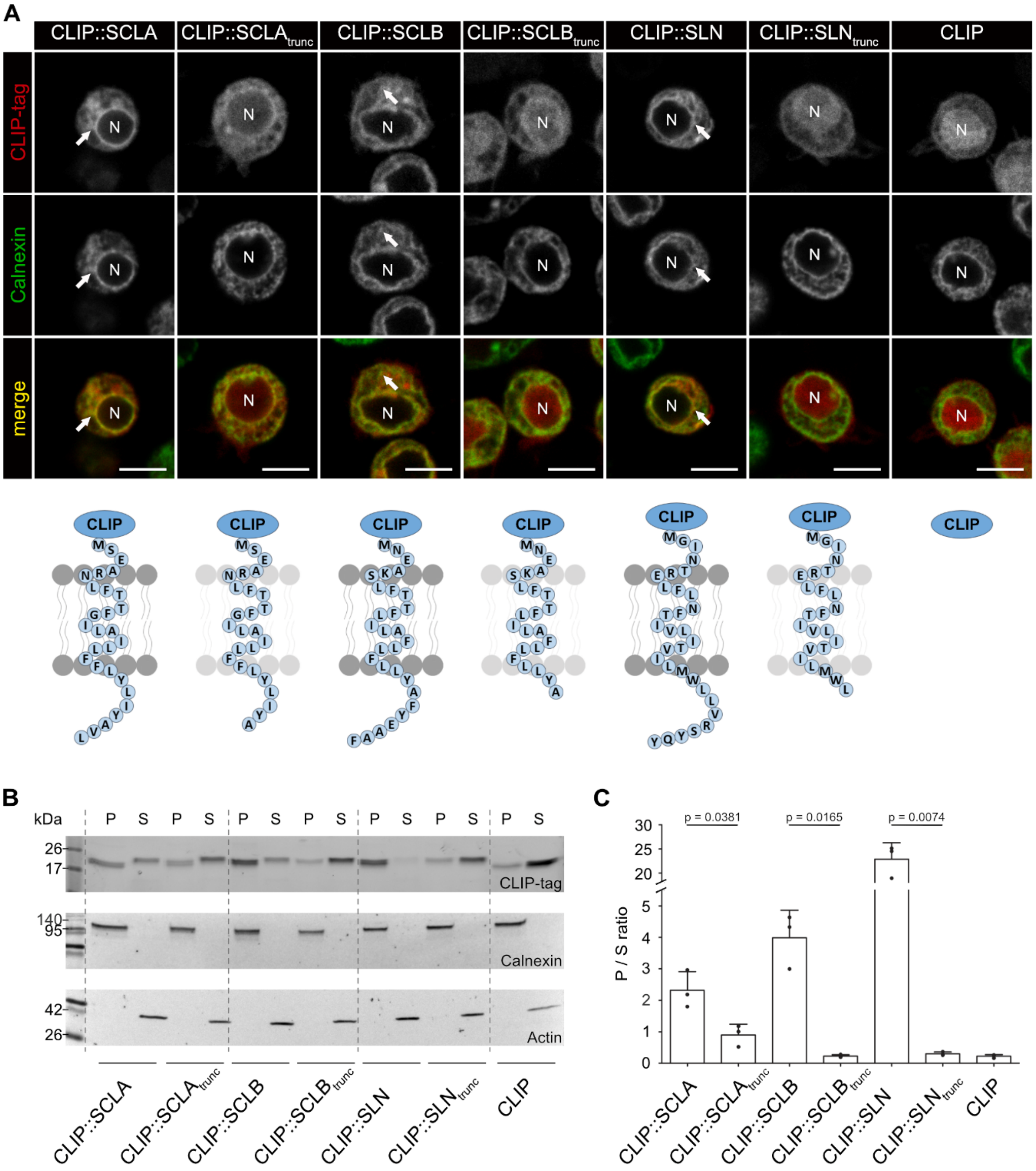

Truncated Sarcolamban and Sarcolipin peptides exhibit reduced membrane anchoring in S2 cells.

(**A**) CLIP-tagged Sarcolamban A (SCLA), Sarcolamban B (SCLB), and Sarcolipin (SLN) were expressed in *Drosophila* S2 cells as full-length (CLIP::SCLA; CLIP::SCLB; CLIP::SLN) and C-terminally truncated constructs (CLIP::SCLA$_{trunc}$; CLIP::SCLB$_{trunc}$; CLIP::SLN$_{trunc}$). All constructs were labeled with CLIP-Cell TMR-Star substrate. Cells expressing the free CLIP-tag were used as a soluble control and anti-Calnexin antibodies (RRID:AB_2722011, 1:200) in combination with anti-mouse-A488 secondary antibodies (RRID:AB_2338845, 1:200) were used as ER marker. While full-length SCL / SLN peptides mainly localize to the ER (arrows), the corresponding truncated peptides accumulate in the nucleus (N). Scale bars: 5 µM. The

lower panel depicts schematics of the analyzed SCL / SLN constructs. (**B**) Subcellular fractions of *Drosophila* S2 cells expressing the indicated CLIP-tagged SCL or SLN constructs were analyzed by SDS-PAGE and subsequent fluorescent in-gel detection of CLIP-tagged fusions. Western blot analysis was performed with anti-Calnexin antibodies (marker for ER membranes) and anti-Actin antibodies (cytosolic marker) to confirm identity of the individual fractions. Coomassie staining (CBB) was used as loading control. P = pellet (membrane-enriched); S = supernatant. (**C**) Peptide-specific ratios between membrane-enriched (P) and soluble (S) fractions were determined by pixel intensity measurements. Depicted are the resultant mean values (+ SD) of three individual biological replicates. Asterisks indicate statistically significant differences between the individual peptide-specific ratios (*$p < 0.05$, **$p < 0.01$, paired t-test, two-tailed). Free CLIP-tag (CLIP) was used as a soluble control. Source data are provided as a Source Data file.

Supplementary figure 7

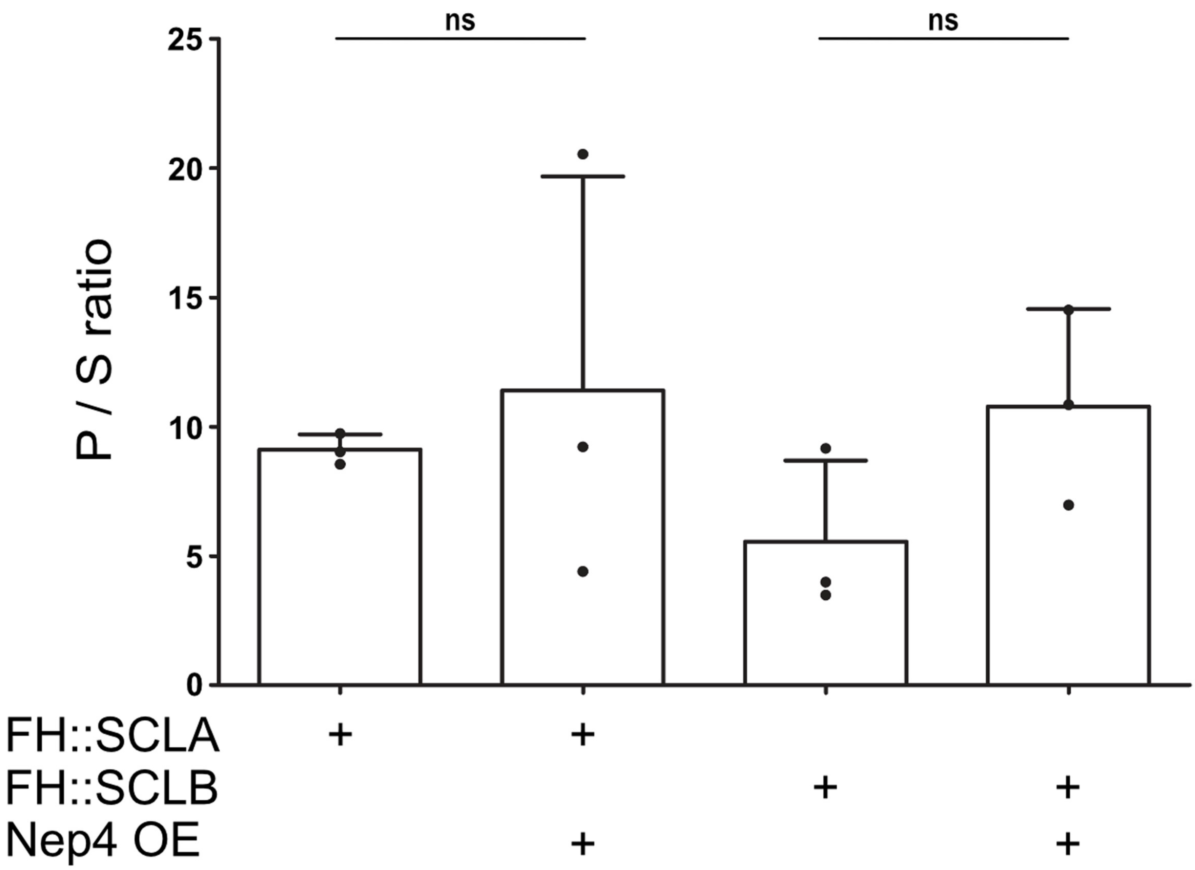

Overexpression of Nep4 does not significantly affect Sarcolamban A or Sarcolamban B membrane localization in third instar larval muscle tissue

Subcellular fractions of *Drosophila* 3rd instar larvae expressing the indicated SCL constructs in a muscle-specific manner (*mef2*-Gal4), with or without co-expression of Nep4, were analyzed by Western blot. Peptide-specific ratios between membrane-enriched (P) and soluble (S) fractions were determined by pixel intensity measurements. Only the monomeric form of the peptides was considered. The diagram depicts the resultant mean values (+ SD) of three individual biological replicates. No statistically significant differences were observed. Source data are provided as a Source Data file.

Supplementary figure 8

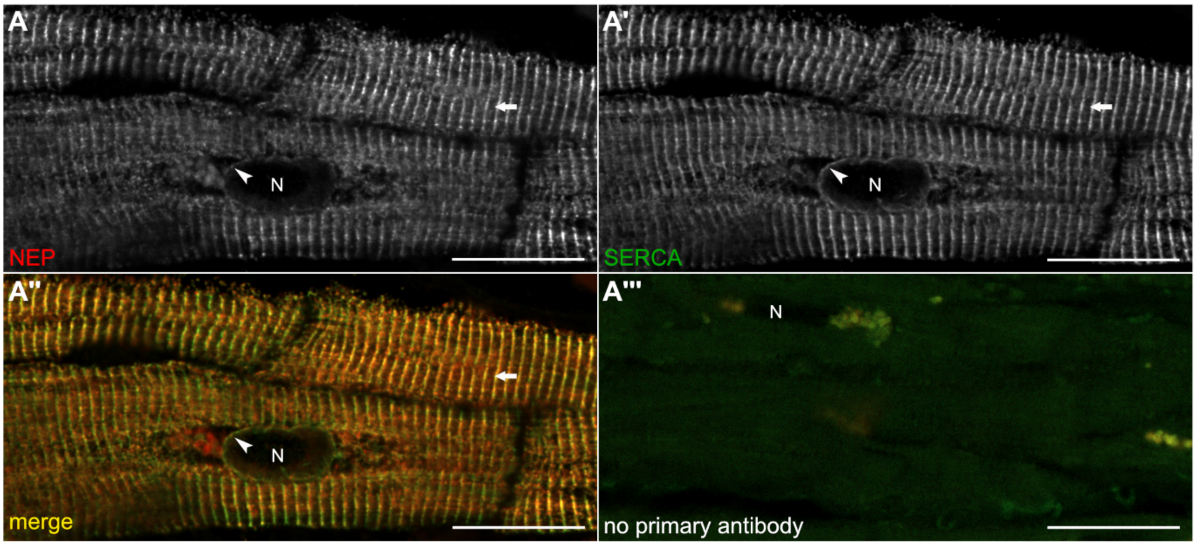

NEP and SERCA partially colocalize in human ventricular cardiomyocytes

(**A**) Human left ventricular cardiomyocytes were stained for NEP (**A**) and SERCA2 (**A´**). Overlapping signals are present along the Z-discs (**A-A´´**, arrow) and in membranes continuous with nuclear membrane (**A-A´´**, arrowhead). In the absence of primary antibodies, no signal above background is visible (**A´´´**). Scale bars: 20 µm.

Supplementary figure 9

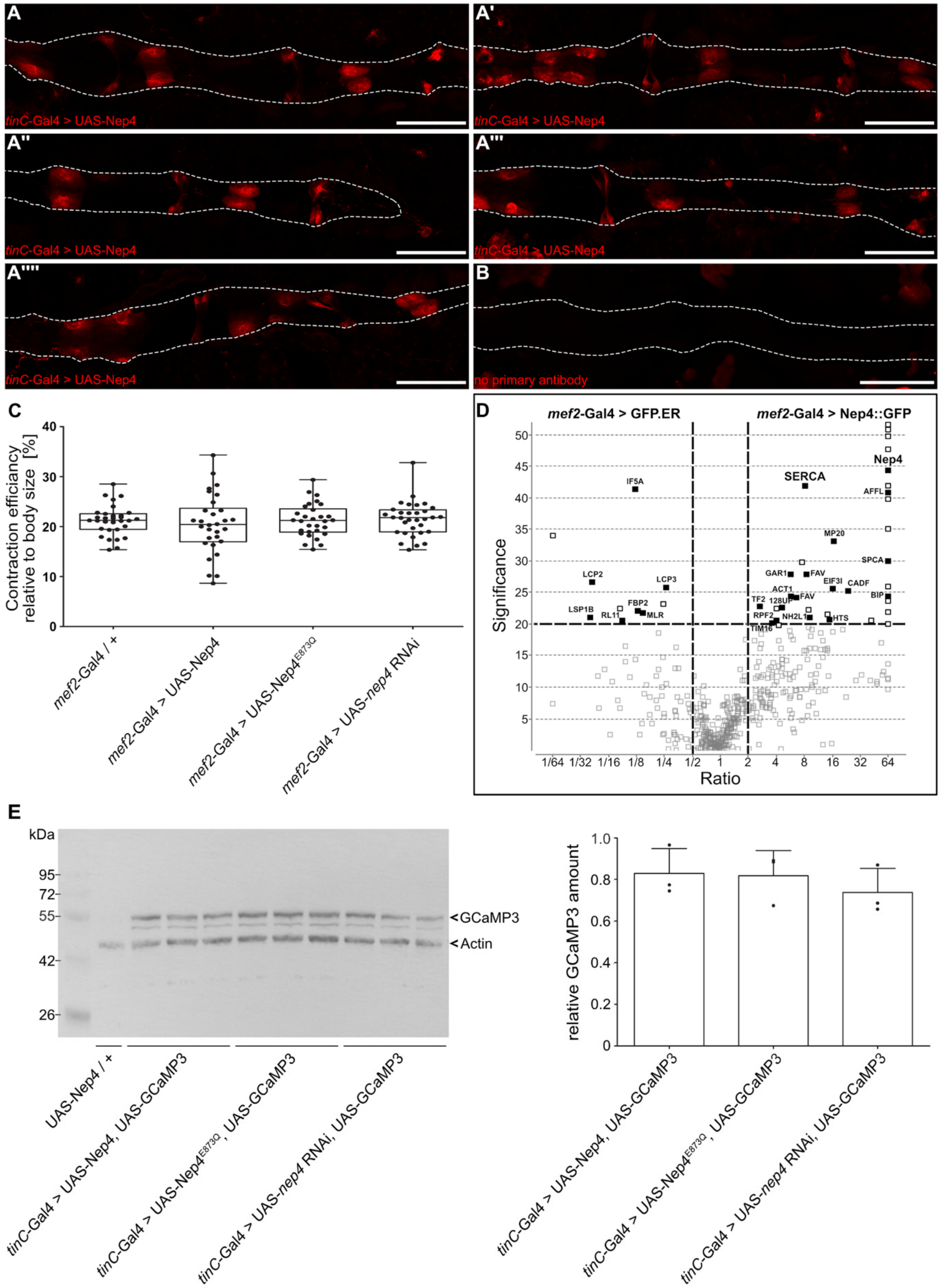

Control data

(**A-A''''**) Immunostainings of one week old adult males with heart-specific overexpression of Nep4 (*tinC*-Gal4 > UAS-Nep4) confirm similar Nep4 protein levels in the individual animals. (**B**) Control stainings lacking primary antibodies do not exhibit any signal above background.

Maximum projections of the heart chamber are shown; scale bars: 100 µm; the heart tube is indicated by dashed lines.

(**C**) Contraction efficiency of third instar larval body wall muscles is not affected by increased Nep4 expression (*mef2*-Gal4 > UAS-Nep4, n = 30), knockdown of *nep4* (*mef2*-Gal4 > UAS-*nep4* RNAi, n = 32) or increased expression of catalytically inactive Nep4 (*mef2*-Gal4 > UAS-Nep4^{E873Q}, n = 30), relative to control animals (*mef2*-Gal4 / +, n = 31). The center line of a boxplot indicates the median; the upper and lower bounds indicate the 75th and 25th percentiles, respectively; and the whiskers indicate the minimum and maximum. Statistical significance was assessed by a one-way ANOVA followed by Dunnett's Multiple Comparison Test.

(**D**) Proteins coprecipitating with Nep4 were identified by pull down assays using total protein extracts of male third instar larvae expressing GFP-tagged Nep4 as bait (*mef2*-Gal4 > Nep4::GFP) and SR-luminal GFP (*mef2*-Gal4 > GFP.ER) as a control. Data are based on three individual biological replicates. Open outlined squares depict proteins with quantification being based on only one detected peptide. Corresponding candidates were excluded from further analyses. A significance value of 20 corresponds to $p < 0.01$ (one-way ANOVA).

(**E**) Representative Western blot of total protein extracts isolated from 1 week old male adult flies of the indicated genotypes. Each lane corresponds to one individual animal. All UAS constructs were expressed in a cardiomyocyte specific manner (*tinC*-Gal4). A control animal lacking GCaMP3 expression (UAS-Nep4 / +) was used to confirm specificity of the Calmodulin antibody (GCaMP3). For GCaMP3 quantification, pixel intensity measurements were normalized to a loading control (Actin). The right panel depicts the mean values (+ SD) of three individual biological replicates. GCaMP3 protein levels are essentially identical in cardiomyocytes of all animals tested and not affected by altered *nep4* expression (one-way ANOVA followed by Tukey´s Multiple Comparison Test). Source data are provided as a Source Data file.

Supplementary Tables

Supplementary table 1

Name	Sequence	Hydrolyzed by	Cleavage products	Cleavage position	Analyzed in
Sarcolamban A_{lum}	YLIYAVLa	Nep4	YLIYA<br>YLIY	A/V<br>Y/A	Fig. 5A
		NEP	YLIYA<br>YLIY<br>*LIYAV / IYAVLa*	A/V<br>Y/A<br>*Y/L; V/L; L/I*	Fig. 5C
Sarcolamban $A_{lum(V6A)}$	YLIYAALa	Nep4	YLIYAA<br>YLIYA<br>LIYAA<br>LIYA	A/L<br>A/A<br>Y/L; A/L<br>Y/L; A/A	Fig. S5A
Sarcolamban B_{lum}	YAFYEAAFa	Nep4	YAFYEAA<br>FYEAAFa<br>YAFYE	A/F<br>A/F<br>E/A	Fig. 5B
		NEP	FYEAAFa<br>*FYEAA / AFYEA*	A/F<br>*A/F; Y/A; A/A*	Fig. 5D
Sarcolamban $B_{lum(F8A)}$	YAFYEAAAa	Nep4	*AFYEAA / FYEAAAa*	*A/A; A/F*	Fig. S5B
Sarcolamban $B_{lum(F3A)}$	YAAYEAAFa	Nep4	AYEAAFa	A/A	Fig. S5C
Sarcolamban $B_{lum(F3A/F8A)}$	YAAYEAAAa	n/a	n/a	n/a	Fig. S5D
Sarcolipin$_{lum}$	WLLVRSYQYa	Nep4	WLLVRSYQ<br>LVRSYQYa<br>WLLVRS<br>WLLVR<br>LVRSY	Q/Y<br>L/L<br>S/Y<br>R/S<br>L/L; Y/Q	Fig. 5F
		NEP	LVRSYQYa<br>WLLVRS<br>WLLVR<br>LVRSY	L/L<br>S/Y<br>R/S<br>L/L; Y/Q	Fig. 5E

Analyzed peptides and cleavage characteristics

Peptides analyzed for *Drosophila* Neprilysin 4 (Nep4) or human Neprilysin (NEP) mediated cleavage. Mutated residues are labelled in red; C-terminal amidation is indicated by an a. Italicized fragments could not be assigned to one unique peptide sequence.

Supplementary table 2

**Cardiac parameter**	***tin*C-Gal4 > UAS-*nep4* RNAi; population**	***tin*C-Gal4 > UAS-*nep4* RNAi; subgroup selected for slower heart rate**	**p values**
Heart rate [bpm]	191.66 (SEM=12.41, n=22)	136.25 (SEM=10.56, n=9)	**0.002**
SR Ca^{2+} load [arb. units]	2.47 (SEM=0.25, n=22)	2.83 (SEM=0.50, n=9)	0.538
SERCA activity [1/sec]	4.11 (SEM=0.53, n=20)	3.40 (SEM=0.76, n=9)	0.564
Constant of relaxation, Tau [sec]	0.20 (SEM=0.02, n=22)	0.26 (SEM=0.04, n=9)	0.314

Variable beating frequencies do not affect Ca^{2+} flux parameters

Depicted are Ca^{2+} flux parameters of *nep4* knockdown flies (*tinC*-Gal4 > UAS-*nep4* RNAi) and of a subgroup of *nep4* knockdown flies selected for slower heart rates. For this subgroup, all effects on SR Ca^{2+} load, SERCA activity, and Tau are consistent with the population data. n depicts the number of analyzed animals. Statistically significant differences are indicated in bold ($p < 0.05$, Students t-test (two-tailed)).
