# Supplementary material for: Neprilysins regulate muscle contraction and heart function via cleavage of SERCA-inhibitory micropeptides
Source: Nat Commun. 2022 Jul 29;13:4420. doi: 10.1038/s41467-022-31974-1 (PMC9338278; doi:10.1038/s41467-022-31974-1)
Supplement: Supplementary file 2 — Description of additional supplementary items [file 41467_2022_31974_MOESM2_ESM.docx]

**Supplementary Movies**

**Supplementary movies 1-4**

*Overexpression and knockdown of* nep4 *affect heart rhythmicity*

Representative heart recordings (30 sec) of adult animals of the following genotypes are shown: *tinC*-Gal4 / + (supplementary movie 1); *tinC*-Gal4 > UAS-Nep4 (supplementary movie 2); *tinC*-Gal4 > UAS-Nep4^E873Q^ (supplementary movie 3); *tinC*-Gal4 > UAS-*nep4* RNAi (supplementary movie 4).
