# Supplementary material for: Neprilysins regulate muscle contraction and heart function via cleavage of SERCA-inhibitory micropeptides
Source: Nat Commun. 2022 Jul 29;13:4420. doi: 10.1038/s41467-022-31974-1 (PMC9338278; doi:10.1038/s41467-022-31974-1)
Supplement: Supplementary file 7 — Reporting Summary [file 41467_2022_31974_MOESM7_ESM.pdf]

nature research

Corresponding author(s): Heiko Meyer
Last updated by author(s): Jun 13, 2022

Reporting Summary

Nature Research wishes to improve the reproducibility of the work that we publish. This form provides structure for consistency and transparency in reporting. For further information on Nature Research policies, see our Editorial Policies and the Editorial Policy Checklist.

Statistics

For all statistical analyses, confirm that the following items are present in the figure legend, table legend, main text, or Methods section.

n/a	Confirmed	
☐	☒	The exact sample size (n) for each experimental group/condition, given as a discrete number and unit of measurement
☐	☒	A statement on whether measurements were taken from distinct samples or whether the same sample was measured repeatedly
☐	☒	The statistical test(s) used AND whether they are one- or two-sided<br>*Only common tests should be described solely by name; describe more complex techniques in the Methods section.*
☒	☐	A description of all covariates tested
☐	☒	A description of any assumptions or corrections, such as tests of normality and adjustment for multiple comparisons
☐	☒	A full description of the statistical parameters including central tendency (e.g. means) or other basic estimates (e.g. regression coefficient) AND variation (e.g. standard deviation) or associated estimates of uncertainty (e.g. confidence intervals)
☐	☒	For null hypothesis testing, the test statistic (e.g. F, t, r) with confidence intervals, effect sizes, degrees of freedom and P value noted<br>*Give P values as exact values whenever suitable.*
☒	☐	For Bayesian analysis, information on the choice of priors and Markov chain Monte Carlo settings
☒	☐	For hierarchical and complex designs, identification of the appropriate level for tests and full reporting of outcomes
☒	☐	Estimates of effect sizes (e.g. Cohen's d, Pearson's r), indicating how they were calculated

Our web collection on statistics for biologists contains articles on many of the points above.

Software and code

Policy information about availability of computer code

Data collection	FireCapture (version 1.2, Torsten Edelmann)<br>Zen (version 2.6, blue edition, Zeiss)<br>FV1000 (version 4.2.1.20, Olympus)<br>Leica Application Suite X (version 3.5.2, LasX)<br>Image Lab Software (version 6.0.1, BioRad)
Data analysis	Matlab R2020a (Update 2, version 9.8.0.1380330, The MathWorks)<br>Semi-automatic Optical Heartbeat Analysis (SOHA, version 3.4.0.0, Oaktree Tech)<br>LabChart 7.0 (AD Instruments, CO, USA)<br>Image Lab software (version 6.0.1, BioRad)<br>ImageJ (version 1.52n, Schneider et al., 2012, PMID: 22930834)<br>FreeStyleTM (version 1.3.115.19, Thermo Fisher Scientific)<br>PEAKS Studio software (version 10.6, Bioinformatics Solutions)<br>GraphPad Prism (version 5.03, GraphPad Software)<br>Affinity Designer (version 1.8.5.703, Serif)

For manuscripts utilizing custom algorithms or software that are central to the research but not yet described in published literature, software must be made available to editors and reviewers. We strongly encourage code deposition in a community repository (e.g. GitHub). See the Nature Research guidelines for submitting code & software for further information.

Data

Policy information about availability of data

All manuscripts must include a data availability statement. This statement should provide the following information, where applicable:

- Accession codes, unique identifiers, or web links for publicly available datasets
- A list of figures that have associated raw data
- A description of any restrictions on data availability

The data supporting the findings from this study are available within the manuscript and its supplementary information. The mass spectrometry proteomics data have been deposited to the ProteomeXchange Consortium via the PRIDE partner repository with the dataset identifier PXD027738 (https://www.ebi.ac.uk/pride/archive/projects/PXD027738). SwissProt database (UP000000803, www.uniprot.org/proteomes/UP000000803) was used to determine peptide-specific amino acid sequences. Source data are provided with this paper.

Field-specific reporting

Please select the one below that is the best fit for your research. If you are not sure, read the appropriate sections before making your selection.

☒ Life sciences ☐ Behavioural & social sciences ☐ Ecological, evolutionary & environmental sciences

For a reference copy of the document with all sections, see nature.com/documents/nr-reporting-summary-flat.pdf

Life sciences study design

All studies must disclose on these points even when the disclosure is negative.

Sample size	Sample sizes were chosen based on established protocols and previous publications. Please see: Fink et al., 2009 (PMID: 19317655), Balcazar et al., 2018 (PMID: 30127403), Tsachaki et al., 2015 (PMID: 25889538), Abrol et al., 2014 (PMID: 25074938)
Data exclusions	To evaluate mass spectrometry based pulldown results, only proteins with quantification being based on two or more detected peptides were considered. Proteins with quantification being based on only one detected peptide were excluded from further analysis.
Replication	To ensure reproducibility, generated datasets were generally based on at least three individual biological replicates (e.g. individual crossings in case of analyzing transgenic Drosophila, or individual transfections in case of corresponding cell culture experiments). All attempts at replication were successful.
Randomization	Animals / cells within the same genotype were randomly allocated to experimental groups and then processed.
Blinding	Investigators were not blind to the group allocation since the experimental design required specific genotypes for experimental and control groups. However, all raw data generated were re-analyzed by at least one additional person on a sample basis.

Reporting for specific materials, systems and methods

We require information from authors about some types of materials, experimental systems and methods used in many studies. Here, indicate whether each material, system or method listed is relevant to your study. If you are not sure if a list item applies to your research, read the appropriate section before selecting a response.

Materials & experimental systems

n/a	Involved in the study	
☐	☒	Antibodies
☐	☒	Eukaryotic cell lines
☒	☐	Palaeontology and archaeology
☐	☒	Animals and other organisms
☐	☒	Human research participants
☒	☐	Clinical data
☒	☐	Dual use research of concern

Methods

n/a	Involved in the study	
☒	☐	ChIP-seq
☒	☐	Flow cytometry
☒	☐	MRI-based neuroimaging

Antibodies

Antibodies used	anti-Nep4 (RRID:AB_2569115, 1:200, Meyer et al., 2009, PMID: 19880729)<br>anti-SERCA (IHC 1:500, WB 1:5000, gift from Mani Ramaswami, Sanyal et al., 2005, PMID: 15520268)<br>anti-SERCA2 (ab219173, 1:100, Abcam Cat# ab219173)<br>anti-NEP (RRID:AB_2894853, 1:100, Abcam Cat# ab256494)<br>anti-GFP (RRID:AB_889471, 1:500, Thermo Fisher Scientific Cat# MA1-952)

anti-GFP (RRID:AB_305564, 1:2000, Abcam Cat# ab6556)
anti-GFP (RRID:AB_300798, 1:750, Abcam Cat# ab13970)
anti-HA (RRID:AB_262051, 1:100, Sigma-Aldrich, Cat# H3663)
anti-Actin (RRID:AB_528068, 1:20, Developmental Studios Hybridoma Bank, Cat# jla20)
anti-Calnexin (RRID:AB_2722011, 1:500, Developmental Studios Hybridoma Bank, Cat# Cnx99A 6-2-1)
anti-Calmodulin (RRID:AB_309644, 1:2000, Millipore Cat# 05-173)
anti-CLIP (RRID:AB_2827567, 1:100, Chromotec Cat# 6f9)

Validation

anti-Nep4 (RRID:AB_2569115): monospecificity confirmed in Meyer et al., 2009, PMID: 19880729

anti-SERCA: monospecificity confirmed in Sanyal et al., 2005, PMID: 15520268

anti-SERCA2 (ab219173): recognizes both reported isoforms (NP_001672.1; NP_733765.1), https://www.abcam.com/serca2-atpase-antibody-ab219173.html

anti-NEP (ab256494): immunogen within Human CD10 aa 50 to the C-terminus. The exact sequence is proprietary (Database link: P08473), knockout validated, https://www.abcam.com/cd10-antibody-epr22867-118-ab256494.html

anti-GFP (RRID:AB_889471): specificity verified by relative expression, https://www.thermofisher.com/antibody/product/eGFP-Monoclonal-Antibody-F56-6A123/MA1-952

anti-GFP (RRID:AB_305564): GFP antibody (ab6556) is reactive against all variants of Aequorea victoria GFP such as S65T-GFP, RS-GFP, YFP, CFP, RFP and EGFP, https://www.abcam.com/gfp-antibodyab6556.html

anti-GFP (RRID:AB_300798): antibody does cross-react with the many fluorescent proteins that are derived from the jellyfish Aequorea victoria. These are all proteins that differ from the original GFP by just a few point mutations (EGFP, YFP, mVenus, CFP, BFP etc.), https://www.abcam.com/gfp-antibody-ab13970.html

anti-HA (RRID:AB_262051): antibody recognizes native as well as denatured-reduced forms of HA-tagged proteins and is reactive with N- or C-terminal HA-tagged fusion proteins, https://www.sigmaaldrich.com/catalog/product/sigma/h3663?lang=en&region=CA

anti-Actin (RRID:AB_528068): antibody recognizes all isoforms of actin over a broad range of species. Specificity was confirmed in Lin et al., 1981, PMID: 7017730, https://dshb.biology.uiowa.edu/JLA20

anti-Calnexin (RRID:AB_2722011): specificity was confirmed in Riedel et al., 2016, PMID: 27256406, https://dshb.biology.uiowa.edu/Cnx99A-6-2-1

anti-Calmodulin (RRID:AB_309644): monospecificity validated for use in WB & IP, specific for both Ca2+-bound and Ca2+-free calmodulin, https://www.merckmillipore.com/DE/de/product/Anti-Calmodulin-Antibody,MM_NF-05-173

anti-CLIP (RRID:AB_2827567): monospecificity was confirmed based on cell extracts from HEK293T cells transiently expressing the protein, https://www.ptglab.com/products/SNAP-CLIP-tag-antibody-6F9.htm?redirect=chromotek#product-information

Eukaryotic cell lines

Policy information about cell lines

Cell line source(s)	Drosophila S2 cells (RRID:CVCL_Z232)<br>Drosophila Sf21 cells (RRID:CVCL_0518)
Authentication	None of the cell lines used were authenticated.
Mycoplasma contamination	All cell lines used in this study were tested for mycoplasma contamination. No mycoplasma contamination was detected.
Commonly misidentified lines<br>(See ICLAC register)	No commonly misidentified cell lines were used in this study.

Animals and other organisms

Policy information about studies involving animals; ARRIVE guidelines recommended for reporting animal research

Laboratory animals

Drosophila melanogaster lines used:

w1118 (RRID:BDSC_5905): one-week-old male F1 offspring were analyzed (Fig. 1, Fig. 2).

mef2-Gal4 (RRID:BDSC_27390): one-week-old male F1 offspring were analyzed in Fig. 1E-F; wandering third instar larvae F1 offspring (male and female) were analyzed in Fig. 3G, Fig. 4, Fig. S2, Fig. S3 and Fig. S4.

tinC-Gal4 (R. Bodmer, Sanford Burnham Medical Research Institute, San Diego, CA, USA): one-week-old male F1 offspring were analyzed in Fig. 1A-D.

	UAS-GCaMP3/UAS-GCaMP3; tinCΔ4-Gal4, UAS-GCaMP3/tinCΔ4-Gal4, UAS-GCaMP3 (Lin et al., 2011): one-week-old male F1 offspring were analyzed in Fig. 2.<br><br>nep4-Gal4 (Meyer et al., 2011; Panz et al., 2012): wandering third instar larvae F1 offspring (male and female) were analyzed in Fig. 3A-F' and Fig. S1.<br><br>UAS-Nep4A (Panz et al., 2012): one-week-old male F1 offspring were analyzed in Fig. 1 and Fig. 2.<br><br>UAS-Nep4-HA (Panz et al., 2012): wandering third instar larvae F1 offspring (male and female) were analyzed in Fig. 3A-F' and Fig. S1.<br><br>UAS-Nep4Ainact (catalytically inactive form (E873Q); Panz et al., 2012): one-week-old male F1 offspring were analyzed in Fig. 1 and Fig. 2.<br><br>v100189 (VDRC): one-week-old male F1 offspring were analyzed in Fig. 1 and Fig. 2.<br><br>UAS-FH::SCLA (Magny et al., 2013): wandering third instar larvae F1 offspring (male and female) were analyzed in Fig. 4, Fig. S2 and Fig. S3.<br><br>UAS-FH::SCLB (Magny et al., 2013): wandering third instar larvae F1 offspring (male and female) were analyzed in Fig. 4, Fig. S2 and Fig. S3.<br><br>UAS-roGFP (this paper): wandering third instar larvae F1 offspring (male and female) were analyzed in Fig. 3G and S4A.<br><br>UAS-Nep4::roGFP (this paper): wandering third instar larvae F1 offspring (male and female) were analyzed in Fig. 3G, Fig. 4, Fig. S2, Fig. S3 and S4B.<br><br>UAS-GFP.ER (RRID:BDSC_59041): wandering third instar larvae F1 offspring (male and female) were used as additional control in pulldown analyses.<br><br>References:<br><br>Lin, N. et al. (2011). A method to measure myocardial calcium handling in adult Drosophila. Circulation research, 108(11), 1306-1315.<br><br>Magny, E. G. et al. (2013). Conserved regulation of cardiac calcium uptake by peptides encoded in small open reading frames. Science, 341(6150), 1116-1120.<br><br>Meyer, H. et al. (2011). Drosophila metalloproteases in development and differentiation: the role of ADAM proteins and their relatives. European journal of cell biology, 90(9), 770-778.<br><br>Panz, M. et al. (2012). A novel role for the non-catalytic intracellular domain of Neprilysins in muscle physiology. Biology of the Cell, 104(9), 553-568.
Wild animals	This study did not involve wild animals.
Field-collected samples	This study did not involve samples collected from the field.
Ethics oversight	The Drosophila work performed in this study did not require any ethical approval or guidance.

Note that full information on the approval of the study protocol must also be provided in the manuscript.

Human research participants

Policy information about studies involving human research participants

Population characteristics	Left ventricular tissue slices were generated from explanted hearts of male patients suffering from ischemic cardiomyopathy (ICM). Age of patients at explantation was 64-66 years and the remaining ejection fraction was 20%-30%.
Recruitment	Left ventricular tissue from three explanted ICM hearts was obtained from the Heart & Diabetes Center NRW, University of Bochum, 32545 Bad Oeynhausen, Germany.
Ethics oversight	The use of human tissue from explanted hearts for research purposes was according to the convention of Helsinki and accepted by the local ethics committee (ethics commission of the faculty of the Ruhr University Bochum, located in Bad Oeynhausen, Germany, vote 21/2013).

Note that full information on the approval of the study protocol must also be provided in the manuscript.
