# Supplementary material for: Neprilysins regulate muscle contraction and heart function via cleavage of SERCA-inhibitory micropeptides
Source: Nat Commun. 2022 Jul 29;13:4420. doi: 10.1038/s41467-022-31974-1 (PMC9338278; doi:10.1038/s41467-022-31974-1)
Supplement: Supplementary file 9 — author list approval form (emails) [file 41467_2022_31974_MOESM9_ESM.pdf]

Dear Ms. Rasheed,

Below please find the approvals of all co-authors along with my original email request. All authors, including myself, agree to the below mentioned changes in authorship.

Best regards,

Heiko Meyer

Dr. Heiko Meyer
University of Osnabrück
Division of Biology/Chemistry
Section of Zoology/Developmental Biology
Barbarastr. 11
49076 Osnabrück, Germany
Tel. +49 541-9692858

Dear co-authors,

with respect to our recent paper ("Neprilysins regulate muscle contraction and heart function via cleavage of SERCA-inhibitory micropeptides", Nature Communications, NCOMMS-21-01535C) I would like to ask for your approval of the following change in authorship:

"Ms Ronja Schiemann and Ms Annika Buhr are now equally-contributing authors."

Thank you for confirming your agreement with these changes via email response to me as soon as possible.

Best regards,

Heiko Meyer

Dr. Heiko Meyer
University of Osnabrück
Division of Biology/Chemistry
Section of Zoology/Developmental Biology
Barbarastr. 11
49076 Osnabrück, Germany
Tel. +49 541-9692858

Von: Ronja Schiemann [mailto:rschiemann@uni-osnabrueck.de]
Gesendet: Samstag, 2. Juli 2022 09:59
An: Dr. Heiko Meyer <heiko.meyer@uni-osnabrueck.de>
Betreff: Re: Fwd: NCOMMS-21-01535C changes in Authorship

Dear Heiko,
I approve the amendment and agree to the change of authorship.

Best regards,
Ronja Schiemann

Von: Annika Buhr [mailto:abuhr@Uni-Osnabrueck.De]
Gesendet: Montag, 4. Juli 2022 11:12
An: 'Dr. Heiko Meyer' <heiko.meyer@uni-osnabrueck.de>
Betreff: RE: NCOMMS-21-01535C changes in Authorship

Dear Heiko,

I agree with the changes in authorship.

Best regards,
Annika Buhr

Von: Eva Cordes [mailto:eva.cordes@uni-osnabrueck.de]
Gesendet: Samstag, 2. Juli 2022 11:57
An: Dr. Heiko Meyer <heiko.meyer@uni-osnabrueck.de>
Betreff: Re: NCOMMS-21-01535C changes in Authorship

Dear Heiko,
thanks for calling me, I agree
Greetings Eva Cordes

Von: Stefan Walter [mailto:Stefan.Walter@uni-osnabrueck.de]
Gesendet: Montag, 4. Juli 2022 09:34
An: 'Dr. Heiko Meyer' <heiko.meyer@uni-osnabrueck.de>
Betreff: AW: NCOMMS-21-01535C changes in Authorship

Dear Heiko,

I hereby declare my consent to the change of authorship.

Regards

Stefan Walter

Von: jheinisc@uni-osnabrueck.de [mailto:jheinisc@uni-osnabrueck.de]
Gesendet: Samstag, 2. Juli 2022 09:07
An: 'Dr. Heiko Meyer' <heiko.meyer@uni-osnabrueck.de>
Betreff: AW: NCOMMS-21-01535C changes in Authorship

Sure, no objections for the change of authorship from my side.
Best

Jürgen Heinisch

Von: Paola Ferrero [mailto:paoferrero@gmail.com]
Gesendet: Samstag, 2. Juli 2022 19:09
An: Heiko Meyer <Heiko.Meyer@uni-osnabrueck.de>
Betreff: Re: NCOMMS-21-01535C changes in Authorship

Hi Heiko,
I agree with the suggested changes in the author list.

Best!

Paola Ferrero

Von: Milting, Hendrik [mailto:HMilting@hdz-nrw.de]
Gesendet: Freitag, 1. Juli 2022 18:20
An: Dr. Heiko Meyer <heiko.meyer@uni-osnabrueck.de>
Betreff: Re: NCOMMS-21-01535C changes in Authorship

Dear Heiko,
No worries from my side.
Best
Hendrik

Von: Achim Paululat [mailto:apaulula@uni-osnabrueck.de]
Gesendet: Montag, 4. Juli 2022 10:53
An: Dr. Heiko Meyer <heiko.meyer@uni-osnabrueck.de>
Betreff: Re: NCOMMS-21-01535C changes in Authorship

Dear Heiko, I agree with the suggested change in authorship.

best regards,

Achim Paululat

Von: sophia.rasheed@nature.com [mailto:sophia.rasheed@nature.com]
Gesendet: Freitag, 1. Juli 2022 16:42
An: heiko.meyer@biologie.uni-osnabrueck.de
Cc: maria-teresa.piccoli@nature.com
Betreff: NCOMMS-21-01535C changes in Authorship

Dear Dr Meyer,

Thank you for sending your revised manuscript to Nature Communications. It has come to our

attention that your most recent author list differs from the one in your original submission. It is Nature Portfolio's policy that any changes to the author list on a paper must be agreed by all of the authors. Please See www.nature.com/authors/editorial_policies/authorship.html for further details.

Please request agreement from all authors including additions and deletions, these can be collected in the two following ways:

1) Approval Form
Using the approval form available here; www.nature.com/documents/nr-author-list-change-form.pdf, arrange for all authors on your paper to sign the statement confirming that they agree to the author list being changed, and then email it back to me.

2) Email consent
Email your co-authors with the change, and ask them to reply to your email confirming that they agree to these changes. Once you have collected these replies, please send all of the co-authors' email responses back to me in one combined file or email (not individually).

Thank you,
Sophia Rasheed
Staff
Nature Communications

This email has been sent through the Springer Nature Tracking System NY-610A-NPG&MTS

Confidentiality Statement:

This e-mail is confidential and subject to copyright. Any unauthorised use or disclosure of its contents is prohibited. If you have received this email in error please notify our Manuscript Tracking System Helpdesk team at http://platformsupport.nature.com .

Details of the confidentiality and pre-publicity policy may be found here http://www.nature.com/authors/policies/confidentiality.html

Privacy Policy | Update Profile
